# Delineating the Role of Various Factors in Renal Disposition of Digoxin through Application of Physiologically Based Kidney Model to Renal Impairment Populations[Fn FN3]

**DOI:** 10.1124/jpet.116.237438

**Published:** 2017-03

**Authors:** Daniel Scotcher, Christopher R. Jones, Aleksandra Galetin, Amin Rostami-Hodjegan

**Affiliations:** Centre for Applied Pharmacokinetic Research, School of Health Sciences, University of Manchester, Manchester, United Kingdom (D.S., A.G., A.R.-H.); DMPK, Oncology iMed, AstraZeneca R&D, Alderley Park, Macclesfield, Cheshire, United Kingdom (C.R.J.); and Simcyp Limited (a Certara Company), Blades Enterprise Centre, Sheffield, United Kingdom (A.R.-H.)

## Abstract

Development of submodels of organs within physiologically-based pharmacokinetic (PBPK) principles and beyond simple perfusion limitations may be challenging because of underdeveloped in vitro-in vivo extrapolation approaches or lack of suitable clinical data for model refinement. However, advantage of such models in predicting clinical observations in divergent patient groups is now commonly acknowledged. Mechanistic understanding of altered renal secretion in renal impairment is one area that may benefit from such models, despite knowledge gaps in renal pathophysiology. In the current study, a PBPK kidney model was developed for digoxin, accounting for the roles of organic anion transporting peptide 4C1 (OATP4C1) and P-glycoprotein (P-gp) in its tubular secretion, with the aim to investigate the impact of age and renal impairment (moderate to severe) on renal drug disposition. Initial PBPK simulations based on changes in glomerular filtration rate (GFR) underestimated the observed reduction in digoxin renal excretion clearance (CL_R_) in subjects with moderately impaired renal function relative to healthy. Reduction in either proximal tubule cell number or the OATP4C1 abundance in the mechanistic kidney model successfully predicted 59% decrease in digoxin CL_R_, in particular when these changes were proportional to reduction in GFR. In contrast, predicted proximal tubule concentration of digoxin was only sensitive to changes in the transporter expression/ million proximal tubule cells. Based on the mechanistic modeling, reduced proximal tubule cellularity and OATP4C1 abundance, and inhibition of OATP4C1-mediated transport, are proposed as possible causes of reduced digoxin renal secretion in renally impaired patients.

## Graphical abstract

**Figure:**
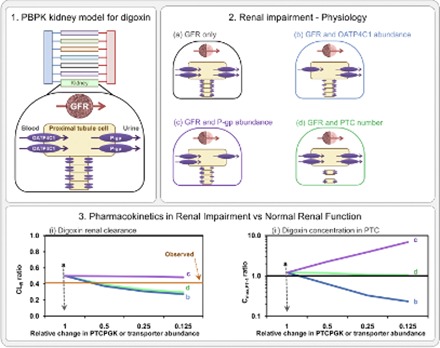

## Introduction

Dosage adjustment is often required in patients with impaired renal function because of the impact this condition may have in altered drug clearance through either decreased renal excretion alone or in combination with reduced metabolism. Ideally, such decisions on dosage adjustment will be supported by recommendations on drug labels that are currently based on statistical analyses of clinical data, often from dedicated but small sized studies (US Food Drug Admin, 2010; Matzke et al., 2011; European Medicines Agency, 2014). The pharmaco-statistical nature of the analysis limits the ability to extrapolate findings beyond the boundaries of the patient groups originally studied. A direct consequence of this, in conjunction with paucity of data in severe renal impairment, has been the large proportion of drug labels void of any recommendations in the most vulnerable renal impairment patients according to recent survey of labels for drugs approved by the Food and Drug Administration in 2013 and 2014 (Jadhav et al., 2015).

A possible solution is the use of physiologically based pharmacokinetic (PBPK) models with embedded organ models that incorporate mechanisms of local disposition. However, these models require careful separation of the drug, system and trial design (Rowland et al., 2011; Rostami-Hodjegan, 2012). In addition, PBPK modeling approaches require case examples to build certainty in their performance. Relatively strong confidence is now placed on the application of PBPK modeling when cytochrome P450 (CYP)-mediated metabolism is the dominant route of elimination by many investigators, including, but not restricted to, the regulatory agencies (Wagner et al., 2015). Conversely, despite recent efforts, evidence for the applicability of PBPK for the prediction of transporter-mediated disposition remains less comprehensive, particularly for the kidney (Zamek-Gliszczynski et al., 2013; Jones et al., 2015; Posada et al., 2015; Varma et al., 2015). The study of Hsu et al., (2014) investigated, through simulation, reduced proximal tubule cellularity as one possible mechanism that could cause changes in renal drug secretion in renal impairment. However, the additional plausible underlying mechanisms, namely reduced transporter expression or inhibition of transporters by uremic solutes, have not been explored. Such simulations are required to assess the implications of specific model assumptions on systemic and local drug concentrations predicted by the PBPK models. In addition, although simulations were performed to investigate changes to proximal tubule intracellular drug concentrations in a transporter mediated drug-drug interaction (DDI) scenario, such changes were not investigated in the renal impairment scenario (Hsu et al., 2014).

This study aimed to develop a PBPK kidney model for digoxin and apply the model to investigate potential effects of age and renal impairment on digoxin renal drug disposition. Rationale for selecting digoxin in this study was the large availability of clinical data to support the estimation and verification of mechanistic kidney model parameters. The initial in vitro-in vivo extrapolation (IVIVE) of digoxin renal excretion clearance (CL_R_) using reported in vitro OATP4C1 transporter intrinsic clearance (CL_int,T_) resulted in under prediction of its CL_R_. Subsequently, this parameter was estimated from collated digoxin clinical data accounting for the uncertainty in the contribution of glomerular filtration to the observed digoxin CL_R_. The developed model was subsequently used to simulate changes in digoxin CL_R_ due to aging and moderate and severe renal impairment. In the case of renal impairment, the impact of reduction in OATP4C1, P-gp abundance per million proximal tubule cells, or proximal tubule cellularity on digoxin CL_R_ and its proximal tubule cell concentrations were investigated.

## Methods

**Fig. 1.**
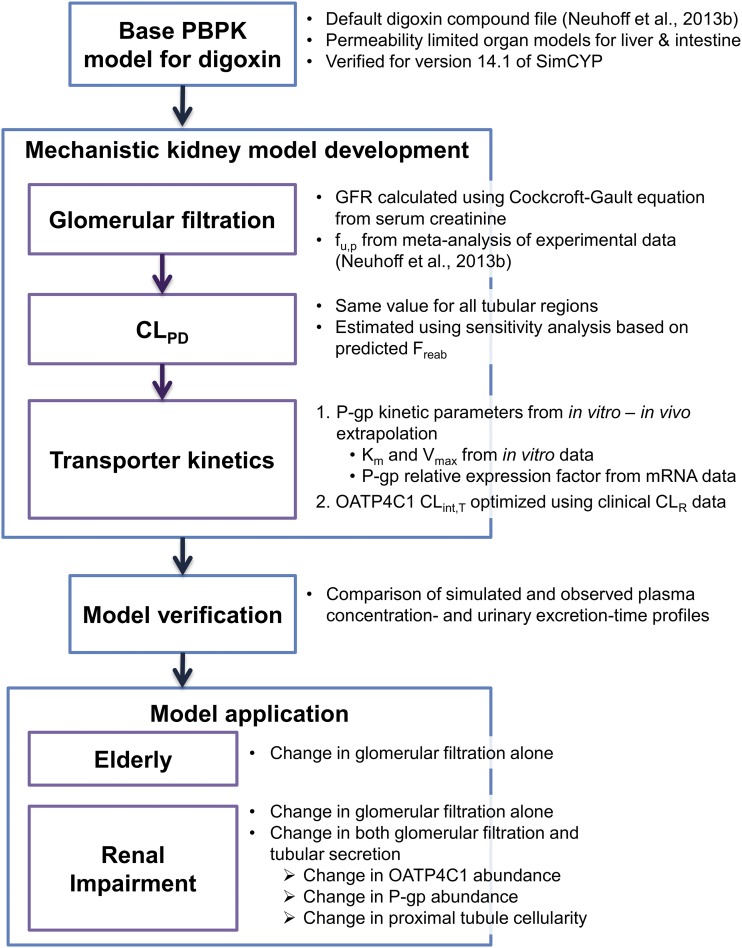
Workflow of the development and application of the PBPK kidney model for digoxin.

The overall strategy for the development and application of the PBPK kidney model for digoxin is presented as a workflow diagram in Fig. 1.

### 

#### Clinical data sources.

Digoxin mean plasma concentration-time profiles and pharmacokinetic parameters were collated from the scientific literature. Pharmacokinetic parameters of interest were the area under the curve (AUC) for the plasma concentration-time profile, the intravenous clearance (CL) and oral clearance (CL/F), volume of distribution at steady state, and CL_R_. Where necessary, data were digitized using GetData Digitizer (version 2, www.getdata-graph-digitizer.com).

#### Verification of basal PBPK model of digoxin.

All simulations presented herein were performed using the SimCYP population-based PBPK simulator software, version 14, release 1 (SimCYP, Sheffield, UK) (Jamei et al., 2009; Jamei et al., 2013). Initial simulations of digoxin plasma concentration-time profiles and CL_R_ were performed using the default “healthy volunteers” population file provided with the SimCYP simulator. Optimized parameters for the full PBPK model of digoxin, including mechanistic models of liver and intestine, were recently published in version 12.2 of the simulator (Neuhoff et al., 2013b). The default compound file provided with version 14.1 of the SimCYP simulator (Supplemental Table S1) was verified against clinical data to ensure consistency between different versions of the software. This verification was performed using several clinical studies, with 10 trials for each set of simulations (Supplemental Table S2) using the default full-PBPK model (Neuhoff et al., 2013b). Upon visual inspection, the simulated concentration-time profiles as well as key pharmacokinetic parameters were not inconsistent with observed data (see Supplemental Fig. S1).

#### IVIVE of renal clearance and optimization of tubular secretion in PBPK model.

Simulation of renal digoxin disposition was performed using the mechanistic kidney model (MechKiM) module in the SimCYP simulator (Neuhoff et al., 2013a). This model comprises eight segments representing the glomerulus, three subregions of proximal tubule, the loop of Henle, the distal tubule, and the cortical and medullary collecting ducts. Separate compartments represent the blood and tubular filtrate of each segment, as well as the tubular cell mass for the seven tubular segments. The model can account for glomerular filtration, passive permeability within all of the tubular segments, and active transport processes within the proximal tubule segments. Readers may refer to previous publications for model equations and assumptions (Hsu et al., 2014; Burt et al., 2016). Digoxin has low passive permeability across cell monolayers, even in the presence of P-gp inhibitors (literature Caco-2 apparent permeability range from 1.15 to 8.03 × 10^−6^ cm/s in various assay formats used (Neuhoff et al., 2003; Zhang and Morris, 2003; Djuv and Nilsen, 2008; Fossati et al., 2008). There are mixed reports from clinical studies suggesting possible urine flow-dependent CL_R_ of digoxin (Steiness, 1974; Halkin et al., 1975; Steiness et al., 1982) and potential role of passive tubular reabsorption in vivo (although minor). Although prediction of tubular reabsorption clearance from physicochemical properties was recently reported, the validated quantitative structure-pharmacokinetic property relationship model does not allow for prediction of passive diffusion clearance (CL_PD_) values in different regions of the nephron (Dave and Morris, 2015). Recently, a static model for prediction of tubular reabsorption was reported that considered regional differences in physiologic parameters for prediction of fraction reabsorbed; this model has not been validated in the MechKiM using appropriate range of model compounds (Scotcher et al., 2016c). To account for tubular reabsorption in the current model, the same value for CL_PD_ parameter (0.01 *µ*l/min/million tubule cells) was assigned to all tubular compartments (i.e., proximal tubule, loop of Henle, distal tubule, and collecting duct). This assigned value for CL_PD_ resulted in simulated fraction reabsorbed for digoxin of 0.12, in agreement with the range predicted using the static model of tubular reabsorption using the Caco-2 apparent permeability literature permeability data (predicted range using static model = 0.064–0.34; see Supplemental Fig. S2) (Scotcher et al., 2016c). The assumption of equal CL_PD_ for each tubular region may underestimate the CL_PD_ in proximal tubule, because its surface area is greater than for the other tubular regions (Scotcher et al., 2016c). Such underestimation of CL_PD_ in proximal tubule could subsequently lead to underestimation of OATP4C1 CL_int,T_. Fraction unbound in kidney cells (f_u,kidney,cell_) of 0.51 was predicted using the Rodgers and Rowland method (Rodgers et al., 2005; Rodgers and Rowland, 2006).

Digoxin secretion in kidney is considered to be mediated predominantly by the OATP4C1 and P-gp transporters, expressed on the basolateral and apical proximal tubule membranes, respectively (Tanigawara et al., 1992; Mikkaichi et al., 2004; He et al., 2014; Lee et al., 2014). A wide range of in vitro *K*_m_ and *V*_max_ values were available for P-gp in the literature, with two studies reporting in vitro data for OATP4C1 (Supplemental Table S3). For consistency, the P-gp *K*_m_ (*µ*M) and *V*_max_ (pmol/min/million cells) parameter values in the mechanistic kidney model were the same as those implemented in the pre-existing mechanistic models for liver and intestine (Troutman and Thakker, 2003; Neuhoff et al., 2013b). For the transporters, sensitivity analyses were performed by modifying the OATP4C1 CL_int,T_ and/or the P-gp relative expression factor (REF) parameters.

P-gp mRNA expression data in kidney and Caco-2 cells were used to inform the REF parameter for this transporter. This inherently assumes that mRNA expression at the tissue level is representative of expression at the cellular level. Lack of quantitative information on differences in P-gp protein expression and activity between different tissues at the cellular level precluded a more mechanistic approach. Because of the lack of in vitro *V*_max_ values reported in the literature, OATP4C1 CL_int,T_ was estimated from reported uptake rate (dX/dt) and initial substrate concentration (C) data (Mikkaichi et al., 2004; Chu et al., 2007), using eq. 1, which assumes that C < < *K*_m_.
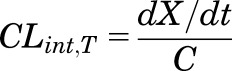
(1)As the transporter expression/abundance data needed to inform the REF scaling factor were lacking, in vitro OATP4C1 CL_int,T_ was normalized to proximal tubule cellularity (Supplemental Table S3), as previously attempted for OCT2 uptake activity (Li et al., 2014). Normalization was performed by assuming equal expression/activity of OATP4C1 in transfected cells and proximal tubule cells and a cellularity of 13 million Madin-Darby canine kidney cells per milligram protein (Richardson et al., 1981). Although large uncertainty is associated with the number of proximal tubule cells per gram of kidney (PTCPGK) in human (Scotcher et al., 2016b), a value of 60 million cells/g kidney was used for this model parameter, in accordance with previous simulation studies (Neuhoff et al., 2013a; Hsu et al., 2014; Li et al., 2014).

As the IVIVE approach outlined above did not successfully predict clinically observed digoxin CL_R_, OATP4C1 CL_int,T_ was estimated using collated digoxin clinical data. First, parameter estimation was performed by fitting the model to the observed plasma concentration profiles of 9 clinical studies after intravenous administration (Johnson and Bye, 1975; Koup et al., 1975; Ochs et al., 1978; Kramer et al., 1979; Ding et al., 2004), using the automated parameter estimation module of the SimCYP simulator. As simultaneous fitting of all data were not possible in the SimCYP software, fitting was performed using each dataset separately, and the overall weighted (by subject number) mean estimate of OATP4C1 CL_int,T_ was subsequently calculated. In addition, the observed overall weighted (by subject number) mean CL_R_ collated from 19 clinical studies in total (Supplemental Table S4) was used to optimize OATP4C1 CL_int,T_ using a detailed sensitivity analysis. In this approach, digoxin pharmacokinetics was simulated using various values of OATP4C1 CL_int,T_ and serum creatinine in a population representative. The OATP4C1 CL_int,T_ that resulted in the simulated CL_R_ in closest agreement with the observed data when serum creatinine was fixed to 80 *µ*mol/l (corresponding to glomerular filtration rate of 120 ml/min in the population representative) was taken forward as the optimal value. This optimization procedure was performed using two separate clinical study designs that had different doses and some differences in the age range. Significant dose-related differences were not expected, because OATP4C1 CL_int,T_ is assumed to be within the linear range of kinetics (Kramer et al., 1979; Rengelshausen et al., 2003). The final model was verified against plasma and urinary digoxin concentration data from additional clinical studies that were not included in the model development (Johnson and Bye, 1975; Ochs et al., 1978; Lindenbaum et al., 1981). In addition, simulation of digoxin plasma concentration data using the final mechanistic model was compared with digoxin PBPK model where mechanistic kidney model was not included and CL_R_ was defined as a single input parameter.

#### Simulation of digoxin CL_R_ in elderly and renal impairment populations.

Digoxin pharmacokinetics was simulated in 100 virtual subjects after intravenous infusion (30 minutes) of 0.75 mg in different virtual populations, with other trial design parameters such as age range and proportion of women of the virtual subjects determined by the general values of the relevant population files. The virtual populations used for simulations included the “Geriatric NEC”, “RenalGFR_30-60”, and “RenalGFR_less_30” populations, which were supplied with the SimCYP simulator. Geriatric NEC population accounts for changes in the age-sex distribution, weights, and heights and kidney size of subjects, whereas the RenalGFR_ populations account for changes in glomerular filtration rate (GFR), protein binding by albumin, hematocrit, kidney weight, and renal blood flow in different stages of renal impairment. There are conflicting reports that digoxin volume of distribution may be altered in patients with end-stage renal disease (Jusko and Weintraub, 1974; Cheng et al., 1997); because of the lack of concordant results on the magnitude of such changes and the fact that these patients were not simulated here, this aspect was not captured in the current study.

In addition the “RenalGFR_30-60” and “RenalGFR_less_30” populations were modified by reducing the value of the PTCPGK parameter. Reducing PTCPGK in the model was investigated as a way to mechanistically represent changes in secretion resulting from tubular cell damage, which may occur during renal impairment and injury (and possibly aging) (Nangaku, 2006; Andreucci et al., 2014; Wang et al., 2014). Alternatively, change in expression of drug transporters per million proximal tubule cells was explored as a mechanism contributing to reduced renal secretion, in line with limited biological data (Naud et al., 2011; Wang and Sweet, 2013). Changes to abundance of the OATP4C1 (basolateral membrane) or P-gp transporters (apical membrane) or PTCPGK were each simulated separately. Although changing the PTCPGK parameter or transporter abundance per million proximal tubule cells for a specific transporter will have the same net effect on overall transporter abundance for that specific transporter per individual (after scaling for kidney weight of the individual), some important differences should be noted. Changes in proximal tubule cell number (i.e., PTCPGK) will affect the overall abundance per kidney of all transporters, as well as scaled CL_PD_ per kidney. The latter will not occur when the transporter abundance per million proximal tubule cells is changed. In addition, the MechKiM model uses the PTCPGK parameter to define the cellularity of all tubular regions, including loop of Henle, distal tubule, and collecting duct compartments. Thus changing the PTCPGK can affect the scaled CL_PD_ in the distal tubular regions and therefore the tubular reabsorption, a factor that is not directly affected by changes to transporter abundance parameters. Reduced abundance of kidney drug transporters per million proximal tubule cells was represented in MechKiM by assigning relative abundances for the OATP4C1 and P-gp transporters in kidney in the “poor transporter” (PT) phenotype as a proportion the SimCYP default “extensive transporter” phenotype value of 1 and setting the frequency of PT in the modified population to 1. Separate changes to PTCPGK or transporter abundance parameters applied equally to each of the three subregion of the proximal tubule. Relative abundance of P-gp in liver and gut remained the same. AUC ratio (AUCR) was calculated using eq. 2:
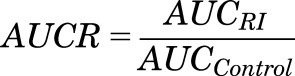
(2)where AUC_RI_ and AUC_Control_ are the mean digoxin AUC in renal impairment subjects (GFR < 60 ml/min/1.73^2^) and mean digoxin AUC in healthy volunteers or patients without renal impairment (GFR > 60 ml/min/1.73^2^). CL_R_ ratio and maximum concentration of digoxin in the cells of the first of the three proximal tubule segments (C_max,PT-1_) ratio were calculated in an analogous manner.

Separately, simulations were performed in the population representative mode after changes in systems parameters in the kidney model, using the “healthy volunteers” population file as a template. In these simulations, digoxin CL_R_ was simulated after changes in GFR either alone or in combination with proportional changes in the OATP4C1 abundance per million proximal tubule cells or PTCPGK parameter (see Table 1 for details).

**TABLE 1:** Parameters used to simulate digoxin CL_R_ Reduction in filtration and secretion was performed to represent changes in renal impairment. Simulated population representative of the “healthy volunteers” population had an age, weight, and body surface area of 20 years, 81 kg, and 1.98 m^2^, respectively. Serum creatinine (input parameter of model) was calculated for each scenario using the Cockcroft-Gault equation (Cockcroft and Gault, 1976), based on the target GFR and the age, weight, and body surface area of the population representative.

GFR	Serum Creatinine Concentration	OATP4C1 Abundance	PTCPGK
*ml/min/1.73 m^2^*	*µmol/l*		*million PTC/g kidney*
136.4*^a^*	76.5	1	60
140	74.5	1.03	61.6
120	86.9	0.88	52.8
100	104.3	0.73	44.0
80	130.4	0.59	35.2
60	173.9	0.44	26.4
40	260.8	0.29	17.6
20	521.7	0.15	8.8
15	695.6	0.11	6.6

^a^ Relative change in GFR for each scenario was calculated using the value of 136.4 ml/ min/m^2^ as baseline and applied to the OATP4C1 abundance or PTCPGK parameter.

## Results

### 

#### Optimization of digoxin kidney transporter kinetic parameters.

**Fig. 2.**
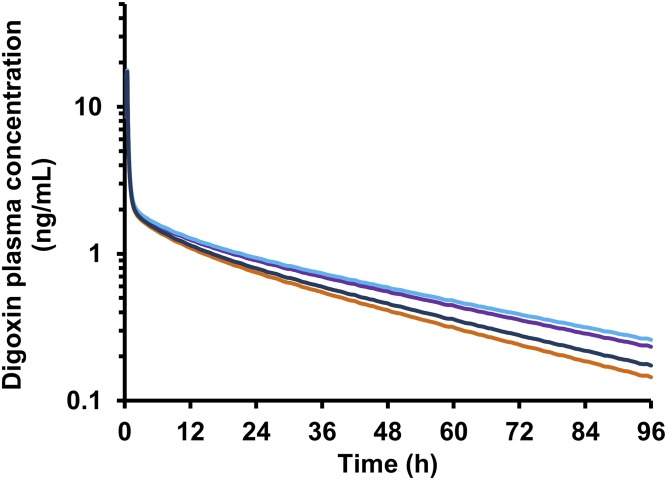
Mean simulated digoxin plasma concentration time profiles (intravenous administration of 1 mg digoxin) for intermediate PBPK models used during development of the mechanistic kidney model. CL_R_ defined by a single input value (136.1 ml/ min) based on the literature analysis (orange line) and CL_R_ simulated using the mechanistic kidney model, accounting for only glomerular filtration (purple line), glomerular filtration and reabsorption (turquoise line), or glomerular filtration, reabsorption, and active secretion (blue line).

The relevance of individual renal mechanisms for digoxin CL_R_ was assessed using the MechKiM module in a stepwise manner. Consideration of either glomerular filtration in isolation or both glomerular filtration and passive tubular reabsorption resulted in simulated digoxin CL_R_ of 98.8 or 87.3 ml/min, respectively, which were both lower than the overall weighted (by subject number) mean observed value of 136.1 ml/min (Supplemental Table S4). Digoxin plasma concentration time profiles for these scenarios are presented in Fig. 2. Simulated AUC_0-∞_ was 29% and 39% higher compared with setting when MechKiM was not activated (i.e., CL_R_ defined using single input parameter) for the "filtration only" and "filtration and reabsorption" scenarios, respectively.

**Fig. 3.**
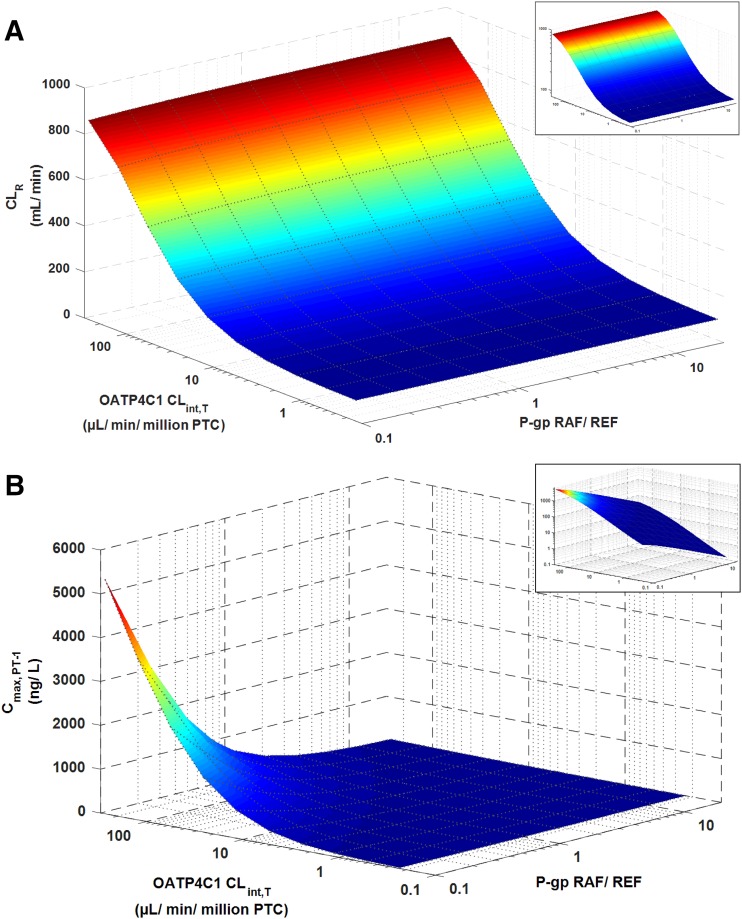
Simulated digoxin CL_R_ (A) and C_max, PT-1_ (B) at different input values for the kidney transporter kinetic parameters. Values of OATP4C1 CL_int,T_ and P-gp REF were varied using the automated sensitivity analysis tool in the SimCYP simulator in a population representative following the clinical trial design reported previously (Greiner et al., 1999). Insets show the graphs presented on logarithmic scales.

The sensitivity of simulated digoxin CL_R_ and AUC to model input parameters was assessed to determine their relative importance. Simulated digoxin CL_R_ and systemic exposure were highly sensitive to changes in OATP4C1 CL_int,T_, in contrast to the marginal effect of changes in P-gp REF (Fig. 3A). Simulated C_max, PT-1_ was sensitive to changes in both OATP4C1 CL_int,T_ and P-gp REF (Fig. 3B). The simulated digoxin CL_R_ and AUC_0-∞_ were insensitive to changes in f_u,kidney,cell_, with minor changes noted at f_u,kidney,cell_ < 0.2 (Supplemental Fig. S3, A and B). C_max, PT-1_ was sensitive to changes in f_u,kidney,cell_ at values below approx. 0.4 (Supplemental Fig. S3C).

Three studies reported P-gp relative mRNA expression between kidney and intestine (Supplemental Table S5). Multiplying these values by the intestine: Caco-2 REF of 2.04 used in the SimCYP gut module resulted in kidney: Caco-2 REFs ranging from 0.78 to 5.34. However, the cellular and tissue expression data were reported in different studies. Therefore, the P-gp REF for Caco-2 cells: kidney used in the final digoxin kidney model (1.51) was based on P-gp mRNA expression data reported in both systems in the same study (Hilgendorf et al., 2007). In vitro OATP4C1-mediated uptake clearance of digoxin was calculated from two uptake rate values reported in the literature (Table 2). In vitro uptake clearance values varied by ∼3 orders of magnitude, and the use of these values to inform the OATP4C1 CL_int,T_ parameter (i.e., IVIVE) resulted in over eightfold difference in simulated CL_R_ (Table 2), representing 67% and 591% of the observed value (Supplemental Table S4).

**TABLE 2:** Values of the OATP4C1 CL_int,T_ parameter estimated by various methods and subsequent simulated digoxin CLR in healthy volunteers using the intravenous trial design from Greiner et al. (1999)

Source of Data	OATP4C1 CL_int,T_ Value	Simulated Digoxin CL_R_
	*µl/min/million PTC*	*ml/min*
In vitro–in vivo extrapolation		
MDCK-OATP4C1 (Mikkaichi et al., 2004)	0.23	90.6
CHO-OATP4C1 (Chu et al., 2007)	270	804.3
Parameter estimation: Fitting to plasma concentration-time profile		
1 mg, i.v. bolus (*n* = 12 subjects) (Kramer et al., 1979)	8.20	
0.01 mg/kg, i.v. 4 min infusion (*n* = 12 subjects) (Rengelshausen et al., 2003)	1.10	
0.5 mg, i.v. 5 min infusion (*n* = 12 subjects) (Ding et al., 2004)	0	
0.75 mg, i.v. bolus (*n* = 8 subjects) (Koup et al., 1975)	0.09	
0.75 mg, i.v. 1 h infusion (*n* = 8 subjects) (Koup et al., 1975)	5.56	
0.75 mg, i.v. 3 min infusion (*n* = 8 subjects) (Johnson and Bye, 1975)	3.30	
0.5 mg, i.v. 1 h infusion (*n* = 9 subjects) (Ochs et al., 1978)	0	
1 mg, i.v. 1 h infusion (*n* = 9 subjects) (Ochs et al., 1978)	0	
1.5 mg, i.v. 1 h infusion (*n* = 9 subjects) (Ochs et al., 1978)	0	
Overall weighted mean ± standard deviation	1.85 ± 2.87	108.8
Parameter estimation: Sensitivity analysis		
Overall weighted mean CL_R_*^a^* (Population representative using trial design 1 mg, i.v. bolus (Kramer et al., 1979))	4.14	133.4
Overall weighted mean CL_R_*^a^* (Population representative using trial design 0.01 mg/ kg, i.v. 4 min infusion (Rengelshausen et al., 2003))	4.14	133.4

PTC, proximal tubule cells; MDCK, Madin-Darby canine kidney.

^a^ CL_R_ data presented in Supplemental Table S4.

**Fig. 4.**
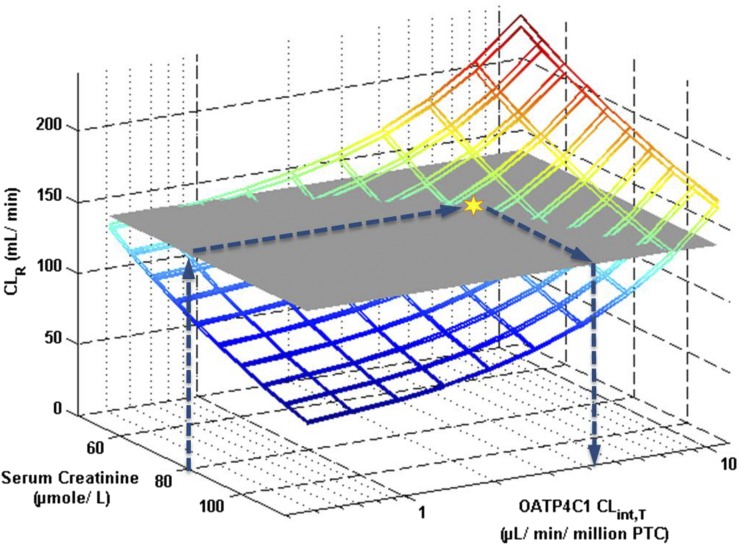
Estimation of OATP4C1 CL_int,T_ parameter using a sensitivity analysis approach by simulating digoxin CL_R_ in population representatives with different serum creatinine values. Colored meshes and gray horizontal plane indicate the simulated CL_R_ and the overall weighted (by subject number) mean CL_R_ obtained from the literature analysis (136.1 ml/min; *n* = 214 healthy subjects), respectively. Values of OATP4C1 CL_int,T_ and serum creatinine parameters were varied using the automated sensitivity analysis tool in the SimCYP simulator. Optimal OATP4C1 value was taken at the intersection (yellow star) of the simulated digoxin CL_R_ with the observed CL_R_ at a serum creatinine value of 80 *µ*mol/l (which corresponds to simulated GFR ∼120 ml/min), as indicated by the blue arrows. Sensitivity analysis was performed twice using clinical trial designs reported previously (Kramer et al., 1979; Rengelshausen et al., 2003).

Alternatively, OATP4C1 CL_int,T_ was obtained by parameter estimation by fitting the model to the observed plasma concentration-time profiles after intravenous administration. Fitting the model separately to the plasma concentration-time profiles from nine clinical studies resulted in weighted mean OATP4C1 CL_int,T_ of 1.85 *µ*l/min/million PTC (Table 2). By using this weighted mean OATP4C1 CL_int,T_ value, simulated digoxin CL_R_ was 80% of the observed value. A sensitivity analysis based approach was next used for estimation of the OATP4C1 CL_int,T_ parameter. The overall weighted mean observed CL_R_ of digoxin obtained from extensive literature search (136.1 ml/min; *n* = 214 healthy subjects) was used as the optimal value (Supplemental Table S4). By using a population representative, digoxin pharmacokinetics were simulated using different OATP4C1 CL_int,T_ and serum creatinine input parameter values. The estimated OATP4C1 CL_int,T_ value, based on the sensitivity analysis approach using a fixed serum creatinine value of 80 *µ*mol/l, was 4.14 *µ*l/min/million PTC (Fig. 4). A serum creatinine value of 80 *µ*mol/l was an assumed average value, because 12 of 19 of the clinical studies used in the literature analysis did not report serum creatinine, creatinine clearance (CL_CR_), or other measurements/estimates of GFR of subjects enrolled. The comparison of simulated (colored mesh) and observed (gray plane) digoxin CL_R_ in Fig. 4 indicates a range of possible values for optimized OATP4C1 CL_int,T_ (i.e., various intersections between simulated and observed CL_R_), depending on the assumed serum creatinine value. From the mean GFR or CL_CR_ data in the 7 digoxin clinical studies that reported these values, the minimum and maximum serum creatinine concentrations (54 and 108 *µ*M) were calculated. Estimation of the OATP4C1 CL_int,T_ parameter assuming these values resulted in twofold differences in the optimized value, as illustrated by Fig. 4. Based on this sensitivity analysis, the estimated OATP4C1 CL_int,T_ value would reliably support conclusions drawn from extrapolation within the context of the current study. All digoxin MechKiM parameters in the developed model are listed in Table 3.

**TABLE 3:** MechKiM parameter values for digoxin model

Description (units)	Value	Comment
f_u,kidney_	0.51	Predicted (Rodgers et al., 2005; Rodgers and Rowland, 2006)
f_u,urine_	1	
OATP4C1 CL_int,T_ (*µ*l/min/million PTC)	4.14	Estimated using sensitivity analysis Allocated to OAT1/ SLC22A6 transporter in MechKiM as OATP4C1 not defined in model
OATP4C1 RAF/ REF	1	
P-gp *K*_m_ (*µ*M)	177	Same as liver/gut (Neuhoff et al., 2013b)
P-gp *V*_max_ (pmol/min/million PTC)	434	Same as liver/gut (Neuhoff et al., 2013b)
P-gp REF	1.51	Calculated from mRNA expression data (Hilgendorf et al., 2007)
CL_PD_ (*µ*l/min/million PTC)	0.01	Estimated using sensitivity analysis and comparing simulated F_reab_ with that predicted using static tubular reabsorption model (Scotcher et al., 2016c) and published Caco-2 data (Neuhoff et al., 2003; Zhang and Morris, 2003; Djuv and Nilsen, 2008; Fossati et al., 2008). Same value for apical and basolateral membranes in all segments of nephron

f_u,kidney_, fraction unbound in kidney; f_u,urine_, fraction unbound in urine; F_reab_, fraction reabsorbed; CL_int,T_, transporter intrinsic clearance; CL_PD_, permeability diffusion clearance; *K*_m_, Michaelis constant; MechKiM, mechanistic kidney model in SimCYP simulator; PTC, proximal tubule cells; RAF, relative activity factor; REF, relative activity factor; *V*_max_, maximal velocity.

**Fig. 5.**
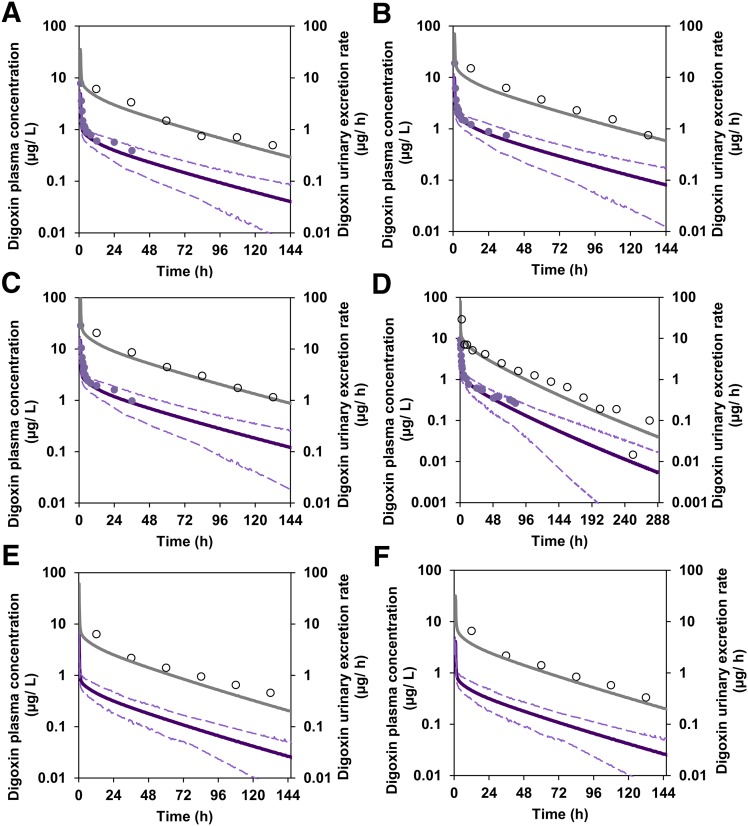
Comparison of simulated and observed digoxin plasma concentration and urinary excretion rate profiles using SimCYP with MechKiM after 0.5 mg by 1 hour infusion (Ochs et al., 1978)(A), 1 mg by 1 hour infusion (Ochs et al., 1978)(B), 1.5 mg by 1 hour infusion (Ochs et al., 1978)(C), 0.75 mg by bolus injection (Johnson and Bye, 1975)(D), and urinary excretion profiles only after 0.4 mg by bolus injection (Lindenbaum et al., 1981)(E) and 0.4 mg by 1 hour infusion (Lindenbaum et al., 1981)(F). Mean (purple solid lines) and 5th and 95th percentiles (dashed purple line) of simulated plasma concentrations are overlaid with mean observed data (purple circles), whereas mean simulated urinary excretion rates (gray solid lines) are overlaid with mean observed data (open circle).

After optimization of the OATP4C1 CL_int,T_ parameter, digoxin pharmacokinetics were then simulated after clinical trial designs reported in studies (Johnson and Bye, 1975; Ochs et al., 1978; Lindenbaum et al., 1981) that were not used in the model development/optimization. Simulated plasma concentration and urinary excretion rate profiles were not inconsistent with the observed data (Fig. 5). In addition, simulated digoxin plasma concentrations were comparable before and after activation of MechKiM to define the CL_R_ of digoxin (Fig. 2).

#### Simulation of digoxin pharmacokinetics in special populations: effects of age and renal impairment.

Mean digoxin CL_R_ simulated in elderly virtual subjects ("Sim-Geriatric NEC" population file) was 90.7 ml/min, 31% lower than simulated CL_R_ in for healthy volunteers (Table 4). This change in digoxin CL_R_ was comparable to the relative change observed in clinical study [36% lower CL_R_ in elderly subjects relative to young subjects (Ewy et al., 1969)], but lower than the relative change in simulated GFR [44% lower in elderly, compared with 54% observed (Ewy et al., 1969)].

**TABLE 4:** Simulated digoxin CL_R_ and AUC_0-∞_ parameters in different virtual populations Each value represents the mean of 100 simulated individuals taken from the virtual population provided with the SimCYP simulator after a single 0.75 mg i.v. dose.

Population	Simulated CL_R_	% of healthy CL_R_	Simulated AUC_0-∞_	% of healthy AUC_0-∞_
	*ml/min*		*µg.min/ml*	
Healthy	131.9	100	3.36	100
Elderly	90.7	69	5.12	152
Moderate renal impairment	65.9	50	5.68	169
Severe renal impairment	43.8	33	7.00	208

**Fig. 6.**
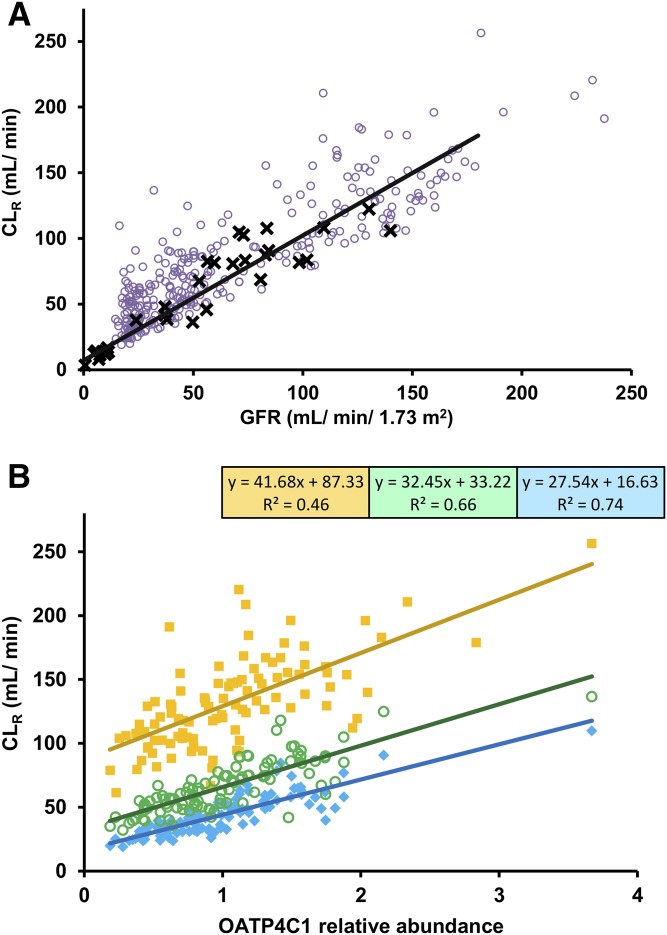
Simulated CL_R_ in comparison with GFR and OATP4C1 abundance in virtual populations. (A) Simulated CL_R_ and GFR data (purple open circle) in healthy and moderate and severe renal impairment virtual subjects, in comparison with reported clinical data (CL_CR_ data on horizontal axis) (black X) (Bloom et al., 1966; Okada et al., 1978). Solid black line represents linear line of best fit using total least squares regression (which recognizes experimental error in both variables) for the observed clinical data. (B) Simulated CL_R_ and GFR in healthy (yellow solid squares) and moderate (green open circles) and severe (turquoise solid diamond) renal impairment virtual subjects. Solid lines represent linear lines of best fit using ordinary least squares regression for data from each simulation, with relevant equations and *R*^2^ shown in boxes.

Mean predicted digoxin CL_R_ in moderate (GFR_30-60) and severe (GFR_less_30) renal impairment virtual populations were 50% and 67% lower than in the simulated healthy volunteers (Table 4). The mean GFR of the virtual subjects with moderate and severe renal impairment were 64% and 82% lower than in the simulated healthy volunteers. Comparison of the observed clinical data with predicted CL_R_ and GFR in the healthy, moderate renal impairment and severe renal impairment virtual populations are shown in Fig. 6A. Although some agreement between predicted and observed data were noted, there was a general trend of overestimation of CL_R_ in the renal impairment populations. The extent of overestimation of CL_R_ was directly correlated with high expression of OATP4C1 in virtual subjects. Average predicted AUCR in moderate renal impairment (1.4) was in agreement with clinical data (1.3). In contrast, model predicted changes in digoxin systemic exposure in severe renal impairment (predicted AUCR of 1.5) were significantly underestimated in comparison with clinical data (AUCR = 3.3) (Okada et al., 1978). GFR and OATP4C1 relative abundance per million proximal tubule cells had similar coefficient of determination (*R*^2^) of the line of best fit with simulated CL_R_ in healthy virtual subjects (Fig. 6B). In contrast, a stronger correlation between either OATP4C1 relative abundance or PTCPGK and CL_R_ was noted in virtual subjects with renal impairment compared with healthy (data not shown).

**Fig. 7.**
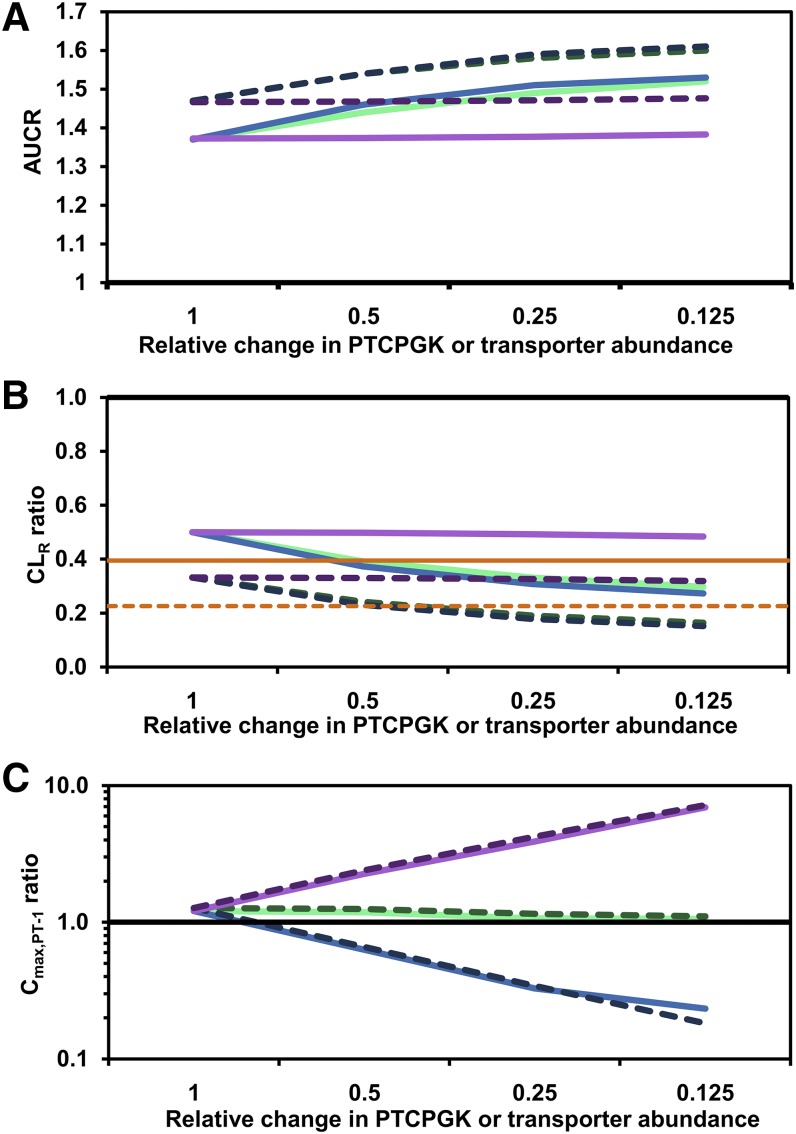
Impact of reduced renal secretion on simulated digoxin AUC ratio (A), CL_R_ (B) and C_max,PT-1_ ratio (C) in renal impairment populations. Renal secretion was reduced either by changing the kidney OATP4C1 or P-gp relative abundance parameters or by reducing the PTCPGK parameter by a proportional amount. Lines represent changes in PTCPGK in moderate renal impairment (light green solid line) and severe renal impairment (green dashed line), OATP4C1 abundance in moderate renal impairment (light blue solid line) and severe renal impairment (blue dashed line), and P-gp abundance in moderate renal impairment (light purple solid line) and severe renal impairment (purple dashed line). Each scenario was simulated in 100 virtual subjects. Solid horizontal black line (ratio = 1) represents the healthy volunteer population; estimated CL_R_ ratios for the average moderate (GFR = 46.5 ml/min/1.73 m^2^; orange solid line) and severe (GFR = 23.5 ml/ min/1.73 m^2^; orange dashed line) renal impairment were calculated based on the correlation of GFR and CL_R_ in the observed data (Bloom et al., 1966; Okada et al., 1978) (A). Relative change of PTCPGK or transporter abundance of 1 indicates that the default moderate or severe renal impairment population in the SimCYP simulator was used

Reduction in OATP4C1 relative abundance per million proximal tubule cells in the renal impairment virtual populations (decrease in REF from 1 to 0.125) or scenario with comparable changes in proximal tubule cell number (7.5–60 million proximal tubule cells/g kidney) both resulted in similar predicted impact on digoxin CL_R_ and AUCR (Fig. 7). For example, maximal reduction in PTCPGK or OATP4C1 abundance per million proximal tubule cells simulated in the severe renal impairment population resulted in predicted CL_R_ ratios of 0.164 or 0.152, respectively (Fig. 7). In contrast to systemic exposure, model assumptions used showed differential effect in the predicted digoxin concentration in the proximal tubule. Reduced PTCPGK had minimal impact on simulated digoxin concentrations in proximal tubule cells, whereas reduced OATP4C1 or P-gp abundance per million proximal tubule cells decreased or increased digoxin intra-cellular concentrations, respectively (Fig. 7).

Equal CL_PD_ was assigned for each tubular region, which may underestimate the CL_PD_ in proximal tubule because of its larger surface area. However, the impact was marginal in the case of digoxin, considering low CL_PD_ in proximal tubule compartment relative to transporter kinetic parameters. Simulations performed to assess the potential impact of underestimation of CL_PD_ in proximal tubule demonstrated that increase in this parameter up to fivefold would have minor overall impact on digoxin CL_R_ and C_max,PT-1_ ratios (Supplemental Fig. S4).

**Fig. 8.**
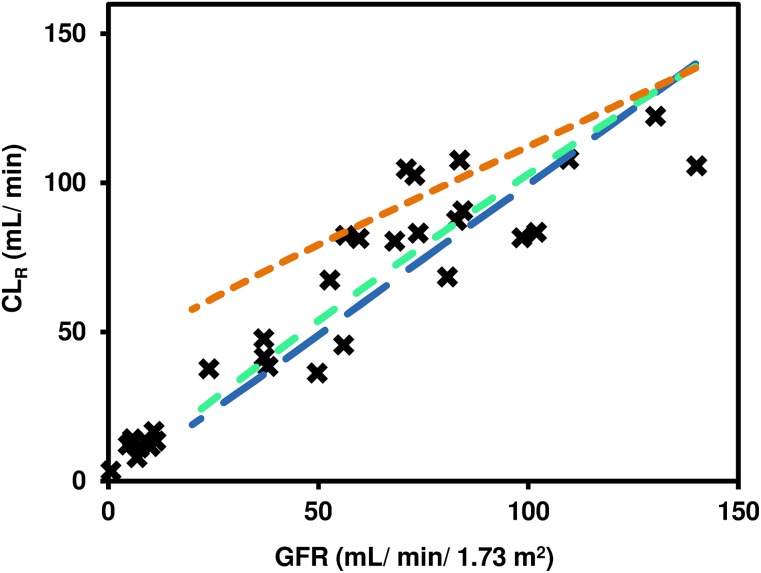
Simulation of digoxin CL_R_ in population representative mode with changes in different systems parameters performed to represent changes in the case of renal impairment. Glomerular filtration rate (range 20–140 ml/min/1.73 m^2^) was changed by altering the serum creatinine parameter (74.5–695.6 *µ*mol/l); OATP4C1 abundance and PTCPGK parameters were altered by a factor proportional to the relative change in GFR from the population representative of the default "healthy volunteers” population (GFR = 136.4 ml/min/1.73 m^2^; serum creatinine = 76.5 *µ*mol/l). Lines represent simulations performed with changes in GFR alone (orange dashed line), both GFR and OATP4C1 abundance (blue dashed line), or both GFR and PTCPGK (green dashed lines). Reported clinical data (black X) are overlaid (Bloom et al., 1966; Okada et al., 1978).

Further simulations were performed in population representative mode, using the "healthy volunteers" population as a template, whereby GFR was altered either alone or alongside proportionally altered OATP4C1 abundance or PTCPGK (Fig. 8). Accounting for changes in tubular secretion in renal impairment, assuming that either OATP4C1 abundance per million proximal tubule cells or PTCPGK are affected proportionally to changes in GFR, resulted in improved agreement between simulated and observed digoxin CL_R_ (Fig. 8).

## Discussion

Use of PBPK kidney models is challenged by a lack of physiological data to inform system parameters and scaling factors, the need for various in vitro data, and availability of suitable clinical data for the drug and population(s) of interest (Scotcher et al., 2016a,b). In an attempt to overcome such challenges in the current study, digoxin was selected as a model drug because of availability of in vitro data and ample clinical data (both plasma and urine) measured in healthy subjects and special populations (e.g., elderly and renal impairment).

Modeling challenges highlighted above are more substantial when considering effects of renal pathophysiology on tubular drug secretion, because the exact contribution of different underlying mechanisms is not fully known. A previous study simulated the impact of reduced proximal tubule cellularity on plasma drug concentrations and CL_R_ (Hsu et al., 2014). However, experimental data on proximal tubule cellularity for healthy and diseased kidneys are unlikely to become available soon, whereas information on inhibitory potential of uremic solutes on renal transporters and transporter abundances are now emerging (Fallon et al., 2016; Hsueh et al., 2016; Prasad et al., 2016). In the current study, after evaluation in the healthy population, a PBPK kidney model for digoxin was applied to investigate the simulated effect of renal impairment on plasma concentrations, CL_R_ and proximal tubule concentrations. Modifications made to different physiological parameters of the model, designed to mimic different underlying mechanisms affecting renal secretion, had divergent effects on output parameters.

### 

#### Development of mechanistic kidney model for digoxin.

A PBPK model for digoxin was previously published and incorporated permeability-limited organ models for the gut and liver (Neuhoff et al., 2013b,c). In the current study the mechanistic kidney model was developed, accounting for the contribution of OATP4C1 and P-gp to digoxin renal secretion (Mikkaichi et al., 2004; Lee et al., 2014). Despite a large amount of available clinical plasma and urine concentration data for digoxin, the P-gp transporter kinetic parameter was practically nonidentifiable because of the lack of measured intracellular digoxin concentrations or sufficiently high temporal resolution of the urinary excretion rate data. As such, accuracy of the IVIVE approach for the P-gp REF parameter could not be assessed, consistent with previous efforts to model renal efflux transport of other drugs (Hsu et al., 2014; Posada et al., 2015). The developed model was therefore unsuitable for simulation of P-gp-mediated digoxin DDIs, although sensitivity analyses indicated that inhibition of renal P-gp would unlikely substantially affect digoxin plasma concentrations (Fig. 3), as proposed by Neuhoff et al. (2013b).

There was also insensitivity of digoxin AUC and CL_R_ to changes in f_u,kidney,cell_ (Supplemental Fig. S3) because of very low CL_PD_ relative to the active transport parameters. Changes to simulated unbound digoxin concentrations in the proximal tubule cells would affect the rate of digoxin diffusion back to the systemic circulation. However, because of low passive permeability, this change was marginal compared with uptake via active transport, and therefore AUC and CL_R_ were mostly unaffected.

Reliable estimate of the OATP4C1 CL_int,T_ could not be obtained based on reported in vitro transporter data and IVIVE scaling factors. Use of in vitro uptake transporter kinetic data from different literature sources resulted in approximately eight-fold difference in predicted CL_R_ (Table 2). This finding further indicates the need for quality in vitro kinetic data and transporter abundance for in vitro systems and human kidney.

The weighted mean OATP4C1 CL_int,T_ obtained by fitting to clinical plasma concentration data were approximately half of the value obtained by using CL_R_ data with a sensitivity analysis (Table 2). The resultant simulated digoxin CL_R_ values were each within 1.5-fold of the observed value. The use of CL_R_ data for optimization was viewed as more reliable because this approach focuses on the parameter of interest with less noise than when using plasma concentration data. Furthermore, separate fitting to mean plasma digoxin concentration data from individual studies was performed rather than global fitting, which can lead to bias in parameter estimation.

Nevertheless, sources of uncertainty of the optimized OATP4C1 CL_int,T_ parameter should be noted. First, the "optimal" CL_R_ value used was obtained from a meta-analysis of clinical data from 19 studies (weighted mean CL_R_ = 136.1 ml/min). This value differed slightly from those obtained in previous literature analyses [151.1 ml/min (Scotcher et al., 2016c) and 160.8 ml/min (Neuhoff et al., 2013b)], although in the current study some data were assigned for model evaluation. Second, the estimated OATP4C1 CL_int,T_ was dependent on the serum creatinine parameter (Fig. 4). Ideally the serum creatinine value would be informed by data obtained from subjects participating in the specific clinical studies, although in the current study, suitable data were missing in the majority of clinical study reports used in the CL_R_ data analysis.

#### Simulation of digoxin renal drug disposition in renal impairment: implications for drug toxicity.

For drugs eliminated predominantly by renal excretion, dosage adjustment (e.g., for elderly patients or with impaired renal function) is informed by the ratio of the estimated GFR (eGFR) or CL_CR_ in patients relative to subjects with normal renal function (Elinder et al., 2014). In the current study the difference in predicted digoxin CL_R_ between elderly and young virtual subjects was smaller (31%) than corresponding differences in simulated GFR [44%; calculated using the Cockcroft-Gault equation (Cockcroft and Gault, 1976)], in agreement with the findings of a clinical study (Ewy et al., 1969). This supports the proposal that despite physiologic changes in kidney during aging (Darmady et al., 1973), proximal tubule secretion is largely retained in elderly subjects without kidney disease (Musso and Oreopoulos, 2011).

Dose adjustment in the clinic tends to use a reduction in renal plasma clearance by a ratio equivalent to the ratio of eGFR (or estimated CL_CR_) in renally impaired patient compared with patient with normal eGFR (Bloom et al., 1966; Okada et al., 1978). This assumes that all processes responsible for renal handling, including tubular secretion, decline in parallel with GFR (Bricker et al., 1960; Naud et al., 2011; Schnaper, 2014). As such, when active secretion occurs, mechanistic models that account only for changes in GFR cannot accurately simulate the decline in drug CL_R_ (Grillo et al., 2012; Hsu et al., 2014; Li et al., 2014). A caveat is that simulated GFR values are sometimes necessarily and inappropriately compared with observed CL_CR_ data (Bauer et al., 1982; Lin et al., 2013).

Various underlying physiological changes have been proposed to cause reduced tubular secretion in renal impairment, including transporter inhibition by uremic solutes, loss of proximal tubule cells, and decreases in transporter expression levels (Naud et al., 2011; Hsueh et al., 2016). Therefore, to mimic renal impairment, changes to transporter abundance (OATP4C1 and P-gp) per million proximal tubule cells and proximal tubule cellularity parameters of the model were considered in the current study. The effect of uremic toxins was not investigated due to limited availability of inhibition data. Equivalent changes to the OATP4C1 abundance or PTCPGK parameters had comparable impact on the predicted AUCR (e.g., reduction of either parameter by 50% in severe renal impairment resulted in 5% increase in AUCR) and CL_R_ ratio (e.g., reducing OATP4C1 abundance per million proximal tubule cells or PTCPGK by 50% in severe renal impairment resulted in 31% or 27% decrease in CL_R_ ratio, respectively). The minor differences between changing OATP4C1 abundance and PTCPGK occurred because the PTCPGK parameter affects also tubular reabsorption, which is not affected by the OATP4C1 abundance parameter (see *Methods*). In contrast, changes in renal P-gp abundance per million proximal tubule cells had negligible effect on these pharmacokinetic parameters for digoxin (Fig. 7).

Conversely, simulated digoxin C_max,PT-1_ was insensitive to changes in proximal tubule cell number but was affected by changes in the transporter abundance parameters (Fig. 7). Overall, the results show that the high degree of correlation between OATP4C1 abundance per million proximal tubule cells and PTCPGK with respect to effect on systemic exposure is not apparent when the dynamic situation within the proximal tubule cell is considered. Therefore, improved understanding of underlying mechanisms behind changes in tubular secretion in renally impaired patients is crucial to determine the increased risk of proximal tubule drug-related toxicity reported in such patients (Naughton, 2008) and for projecting the combined impact of multiple factors (e.g., transporter mediated DDIs in renally impaired subjects).

### Conclusion

A mechanistic kidney model for digoxin was developed accounting for the roles of OATP4C1 and P-gp in its tubular secretion and subsequently applied for the prediction of digoxin pharmacokinetics in special populations. Consideration of reduced GFR in renal impairment in isolation was insufficient to capture changes in digoxin CL_R_ in this patient group. Different mechanisms associated with reduced active tubular secretion in renal impairment were explored in the kidney model, namely reduced transporter abundance per million proximal tubule cells and decrease in proximal tubule cellularity. Although quantitative transporter abundance data in normal human kidney samples are emerging and are likely to become available for diseased tissue, data on proximal tubule cellularity in normal and diseased kidneys are lacking. Reduction in OATP4C1 expression or PTCPGK each caused comparable changes on the predicted digoxin systemic exposure and CL_R_. In contrast, predicted proximal tubule concentration of digoxin was only sensitive to changes in the transporter expression parameters. These results suggest that depending on the output parameter of interest, accurate model specification of pathophysiology may or may not be important. However, the implications of potential misspecification could be more severe if the model developed is applied to extrapolate to more complex scenarios (e.g., transporter-mediated DDIs in renally impaired patients).

## Abbreviations

AUC: area under the curve
AUCR: area under the curve ratio
CL: clearance
CL_CR_: creatinine clearance
CL/F: oral clearance
CL_int,T_: transporter intrinsic clearance
CL_PD_: passive diffusion clearance
CL_R_: renal excretion clearance
C_max,PT-1_: maximum concentration in proximal tubule segment 1 cells
CYP: cytochrome P450
DDI: drug-drug interaction
eGFR: estimated glomerular filtration rate
f_u_,_kidney,cell_: fraction unbound in kidney cells
GFR: glomerular filtration rate
IVIVE: in vitro-in vivo extrapolation
MechKiM: mechanistic kidney model
OATP: organic anion transporting peptide
PBPK: physiologically based pharmacokinetic
P-gp: P-glycoprotein
PT: “poor transporter” phenotype
PTC: proximal tubule cells
PTCPGK: proximal tubule cells per gram kidney
REF: relative expression factor
V_ss_: volume of distribution at steady state

## References

[B1] Andreucci M Faga T Pisani A Sabbatini M Russo D Michael A (2014) Prevention of contrast-induced nephropathy through a knowledge of its pathogenesis and risk factors. Sci World J 2014:823169. 10.1155/2014/823169 PMC4266998 25525625

[B2] Bauer JH Brooks CS Burch RN (1982) Clinical appraisal of creatinine clearance as a measurement of glomerular filtration rate. Am J Kidney Dis 2:337–346. 7148824 10.1016/s0272-6386(82)80091-7

[B3] Bloom PM Nelp WB Tuell SH (1966) Relationship of the excretion of tritiated digoxin to renal function. Am J Med Sci 251:133–144. 5904731 10.1097/00000441-196602000-00002

[B4] Bricker NS Morrin PA Kime SW Jr (1960) The pathologic physiology of chronic Bright’s disease. An exposition of the “intact nephron hypothesis”. Am J Med 28:77–98. 13804370 10.1016/0002-9343(60)90225-4

[B5] Burt HJ Neuhoff S Almond L Gaohua L Harwood MD Jamei M Rostami-Hodjegan A Tucker GT Rowland-Yeo K (2016) Metformin and cimetidine: Physiologically based pharmacokinetic modelling to investigate transporter mediated drug-drug interactions. Eur J Pharm Sci 88:70–82. 27019345 10.1016/j.ejps.2016.03.020

[B6] Cheng JW Charland SL Shaw LM Kobrin S Goldfarb S Stanek EJ Spinler SA (1997) Is the volume of distribution of digoxin reduced in patients with renal dysfunction? Determining digoxin pharmacokinetics by fluorescence polarization immunoassay. Pharmacotherapy 17:584–590. 9165563

[B7] Chu XY Bleasby K Yabut J Cai X Chan GH Hafey MJ Xu S Bergman AJ Braun MP Dean DC (2007) Transport of the dipeptidyl peptidase-4 inhibitor sitagliptin by human organic anion transporter 3, organic anion transporting polypeptide 4C1, and multidrug resistance P-glycoprotein. J Pharmacol Exp Ther 321:673–683. 17314201 10.1124/jpet.106.116517

[B8] Cockcroft DW Gault MH (1976) Prediction of creatinine clearance from serum creatinine. Nephron 16:31–41. 1244564 10.1159/000180580

[B9] Darmady EM Offer J Woodhouse MA (1973) The parameters of the ageing kidney. J Pathol 109:195–207. 4719771 10.1002/path.1711090304

[B10] Dave RA Morris ME (2015) Quantitative structure-pharmacokinetic relationships for the prediction of renal clearance in humans. Drug Metab Dispos 43:73–81. 25352657 10.1124/dmd.114.059857 PMC4279087

[B11] Ding R Tayrouz Y Riedel KD Burhenne J Weiss J Mikus G Haefeli WE (2004) Substantial pharmacokinetic interaction between digoxin and ritonavir in healthy volunteers. Clin Pharmacol Ther 76:73–84. 15229466 10.1016/j.clpt.2004.02.008

[B12] Djuv A Nilsen OG (2008) Caco-2 cell methodology and inhibition of the P-glycoprotein transport of digoxin by Aloe vera juice. Phytother Res 22:1623–1628. 19003953 10.1002/ptr.2536

[B13] Elinder C-G Bárány P Heimbürger O (2014) The use of estimated glomerular filtration rate for dose adjustment of medications in the elderly. Drugs Aging 31:493–499. 24902935 10.1007/s40266-014-0187-z

[B14] European Medicines Agency (2014) Guideline on the Evaluation of the Pharmacokinetics of Medicinal Products in Patients with Decreased Renal Function (CHMP/EWP/225/02). Committee for Human Medicinal Products, CHMP, London.

[B15] Ewy GA Kapadia GG Yao L Lullin M Marcus FI (1969) Digoxin metabolism in the elderly. Circulation 39:449–453. 5778245 10.1161/01.cir.39.4.449

[B16] Fallon JK Smith PC Xia CQ Kim M-S (2016) Quantification of four efflux drug transporters in liver and kidney across species using targeted quantitative proteomics by isotope dilution nanoLC-MS/MS. Pharm Res 33:2280–2288. 27356525 10.1007/s11095-016-1966-5

[B17] Fossati L Dechaume R Hardillier E Chevillon D Prevost C Bolze S Maubon N (2008) Use of simulated intestinal fluid for Caco-2 permeability assay of lipophilic drugs. Int J Pharm 360:148–155. 18539418 10.1016/j.ijpharm.2008.04.034

[B18] Greiner B Eichelbaum M Fritz P Kreichgauer H-P von Richter O Zundler J Kroemer HK (1999) The role of intestinal P-glycoprotein in the interaction of digoxin and rifampin. J Clin Invest 104:147–153. 10411543 10.1172/JCI6663 PMC408477

[B19] Grillo JA Zhao P Bullock J Booth BP Lu M Robie-Suh K Berglund EG Pang KS Rahman A Zhang L (2012) Utility of a physiologically-based pharmacokinetic (PBPK) modeling approach to quantitatively predict a complex drug-drug-disease interaction scenario for rivaroxaban during the drug review process: implications for clinical practice. Biopharm Drug Dispos 33:99–110. 22270945 10.1002/bdd.1771

[B20] Halkin H Sheiner LB Peck CC Melmon KL (1975) Determinants of the renal clearance of digoxin. Clin Pharmacol Ther 17:385–394. 1122680 10.1002/cpt1975174385

[B21] He J Yu Y Prasad B Chen X Unadkat JD (2014) Mechanism of an unusual, but clinically significant, digoxin-bupropion drug interaction. Biopharm Drug Dispos 35:253–263. 24436229 10.1002/bdd.1890

[B22] Hilgendorf C Ahlin G Seithel A Artursson P Ungell AL Karlsson J (2007) Expression of thirty-six drug transporter genes in human intestine, liver, kidney, and organotypic cell lines. Drug Metab Dispos 35:1333–1340. 17496207 10.1124/dmd.107.014902

[B23] Hsu V de L T Vieira M Zhao P Zhang L Zheng JH Nordmark A Berglund EG Giacomini KM Huang SM (2014) Towards quantitation of the effects of renal impairment and probenecid inhibition on kidney uptake and efflux transporters, using physiologically based pharmacokinetic modelling and simulations. Clin Pharmacokinet 53:283–293. 24214317 10.1007/s40262-013-0117-y PMC3927056

[B24] Hsueh C-H Yoshida K Zhao P Meyer TW Zhang L Huang S-M Giacomini KM (2016) Identification and quantitative assessment of uremic solutes as inhibitors of renal organic anion transporters, OAT1 and OAT3. Mol Pharm 13:3130–3140. 27467266 10.1021/acs.molpharmaceut.6b00332

[B25] Jadhav PR Cook J Sinha V Zhao P Rostami-Hodjegan A Sahasrabudhe V Stockbridge N Powell JR (2015) A proposal for scientific framework enabling specific population drug dosing recommendations. J Clin Pharmacol 55:1073–1078. 26109076 10.1002/jcph.579

[B26] Jamei M Marciniak S Edwards D Wragg K Feng K Barnett A Rostami-Hodjegan A (2013) The simcyp population based simulator: architecture, implementation, and quality assurance. In Silico Pharmacol 1:9. 25505654 10.1186/2193-9616-1-9 PMC4230310

[B27] Jamei M Marciniak S Feng K Barnett A Tucker G Rostami-Hodjegan A (2009) The Simcyp population-based ADME simulator. Expert Opin Drug Metab Toxicol 5:211–223. 19199378 10.1517/17425250802691074

[B28] Johnson BF Bye C (1975) Maximal intestinal absorption of digoxin, and its relation to steady state plasma concentration. Br Heart J 37:203–208. 1091283 10.1136/hrt.37.2.203 PMC484102

[B29] Jones HM Chen Y Gibson C Heimbach T Parrott N Peters SA Snoeys J Upreti VV Zheng M Hall SD (2015) Physiologically based pharmacokinetic modeling in drug discovery and development: a pharmaceutical industry perspective. Clin Pharmacol Ther 97:247–262. 25670209 10.1002/cpt.37

[B30] Jusko WJ Weintraub M (1974) Myocardial distribution of digoxin and renal function. Clin Pharmacol Ther 16:449–454. 4414761 10.1002/cpt1974163part1449

[B31] Koup JR Greenblatt DJ Jusko WJ Smith TW Koch-Weser J (1975) Pharmacokinetics of digoxin in normal subjects after intravenous bolus and infusion doses. J Pharmacokinet Biopharm 3:181–192. 1159622 10.1007/BF01067907

[B32] Kramer WG Kolibash AJ Lewis RP Bathala MS Visconti JA Reuning RH (1979) Pharmacokinetics of digoxin: relationship between response intensity and predicted compartmental drug levels in man. J Pharmacokinet Biopharm 7:47–61. 458556 10.1007/BF01059440

[B33] Lee CA Kalvass JC Galetin A Zamek-Gliszczynski MJ (2014) ITC commentary on the prediction of digoxin clinical drug-drug interactions from in vitro transporter assays. Clin Pharmacol Ther 96:298–301. 25141954 10.1038/clpt.2014.94

[B34] Li J Kim S Sha X Wiegand R Wu J LoRusso P (2014) Complex disease-, gene-, and drug-drug interactions: impacts of renal function, CYP2D6 phenotype, and OCT2 activity on veliparib pharmacokinetics. Clin Cancer Res 20:3931–3944. 24947923 10.1158/1078-0432.CCR-14-0791 PMC4151156

[B35] Lin YC Bansal N Vittinghoff E Go AS Hsu CY (2013) Determinants of the creatinine clearance to glomerular filtration rate ratio in patients with chronic kidney disease: a cross-sectional study. BMC Nephrol 14:268. 24305166 10.1186/1471-2369-14-268 PMC3924195

[B36] Lindenbaum J Long R Wenger T Mallis G Cato A (1981) Lack of difference in digoxin urinary excretion with two intravenous infusion rates. Clin Pharmacol Ther 30:317–320. 7273595 10.1038/clpt.1981.166

[B37] Matzke GR Aronoff GR Atkinson AJ Jr Bennett WM Decker BS Eckardt K-U Golper T Grabe DW Kasiske B Keller F (2011) Drug dosing consideration in patients with acute and chronic kidney disease-a clinical update from Kidney Disease: Improving Global Outcomes (KDIGO). Kidney Int 80:1122–1137. 21918498 10.1038/ki.2011.322

[B38] Mikkaichi T Suzuki T Onogawa T Tanemoto M Mizutamari H Okada M Chaki T Masuda S Tokui T Eto N (2004) Isolation and characterization of a digoxin transporter and its rat homologue expressed in the kidney. Proc Natl Acad Sci USA 101:3569–3574. 14993604 10.1073/pnas.0304987101 PMC373503

[B39] Musso CG Oreopoulos DG (2011) Aging and physiological changes of the kidneys including changes in glomerular filtration rate. Nephron, Physiol 119 (Suppl 1):1–5. 10.1159/000328010 21832859

[B40] Nangaku M (2006) Chronic hypoxia and tubulointerstitial injury: a final common pathway to end-stage renal failure. J Am Soc Nephrol 17:17–25. 16291837 10.1681/ASN.2005070757

[B41] Naud J Michaud J Beauchemin S Hébert M-J Roger M Lefrancois S Leblond FA Pichette V (2011) Effects of chronic renal failure on kidney drug transporters and cytochrome P450 in rats. Drug Metab Dispos 39:1363–1369. 21525170 10.1124/dmd.111.039115

[B42] Naughton CA (2008) Drug-induced nephrotoxicity. Am Fam Physician 78:743–750. 18819242

[B43] Neuhoff S Gaohua L Burt H Jamei M Li L Tucker GT Rostami-Hodjegan A (2013 a) Accounting for transporters in renal clearance: towards a mechanistic kidney model (Mech KiM), in Transporters in Drug Development (Sugiyama Y Steffansen B, eds) pp 155–177, Springer, New York.

[B44] Neuhoff S Ungell A-L Zamora I Artursson P (2003) pH-dependent bidirectional transport of weakly basic drugs across Caco-2 monolayers: implications for drug-drug interactions. Pharm Res 20:1141–1148. 12948010 10.1023/a:1025032511040

[B45] Neuhoff S Yeo KR Barter Z Jamei M Turner DB Rostami-Hodjegan A (2013 b) Application of permeability-limited physiologically-based pharmacokinetic models: part I-digoxin pharmacokinetics incorporating P-glycoprotein-mediated efflux. J Pharm Sci 102:3145–3160. 23703021 10.1002/jps.23594

[B46] Neuhoff S Yeo KR Barter Z Jamei M Turner DB Rostami-Hodjegan A (2013 c) Application of permeability-limited physiologically-based pharmacokinetic models: part II - prediction of P-glycoprotein mediated drug-drug interactions with digoxin. J Pharm Sci 102:3161–3173. 23686764 10.1002/jps.23607

[B47] Ochs HR Greenblatt DJ Bodem G Harmatz JS (1978) Dose-independent pharmacokinetics of digoxin in humans. Am Heart J 96:507–511. 358815 10.1016/0002-8703(78)90162-x

[B48] Okada RD Hager WD Graves PE Mayersohn M Perrier DG Marcus FI (1978) Relationship between plasma concentration and dose of digoxin in patients with and without renal impairment. Circulation 58:1196–1203. 709776 10.1161/01.cir.58.6.1196

[B49] Posada MM Bacon JA Schneck KB Tirona RG Kim RB Higgins JW Pak YA Hall SD Hillgren KM (2015) Prediction of renal transporter mediated drug-drug interactions for pemetrexed using physiologically based pharmacokinetic modeling. Drug Metab Dispos 43:325–334. 25504564 10.1124/dmd.114.059618

[B50] Prasad B Johnson K Billington S Lee C Chung GW Brown CD Kelly EJ Himmelfarb J Unadkat JD (2016) Abundance of drug transporters in the human kidney cortex as quantified by quantitative targeted proteomics. Drug Metab Dispos 44:1920–1924 10.1124/dmd.116.072066. 27621205 10.1124/dmd.116.072066 PMC5118637

[B51] Rengelshausen J Göggelmann C Burhenne J Riedel KD Ludwig J Weiss J Mikus G Walter-Sack I Haefeli WE (2003) Contribution of increased oral bioavailability and reduced nonglomerular renal clearance of digoxin to the digoxin-clarithromycin interaction. Br J Clin Pharmacol 56:32–38. 12848773 10.1046/j.1365-2125.2003.01824.x PMC1884337

[B52] Richardson JC Scalera V Simmons NL (1981) Identification of two strains of MDCK cells which resemble separate nephron tubule segments. Biochim Biophys Acta 673:26–36. 6110442

[B53] Rodgers T Leahy D Rowland M (2005) Physiologically based pharmacokinetic modeling 1: predicting the tissue distribution of moderate-to-strong bases. J Pharm Sci 94:1259–1276. 15858854 10.1002/jps.20322

[B54] Rodgers T Rowland M (2006) Physiologically based pharmacokinetic modelling 2: predicting the tissue distribution of acids, very weak bases, neutrals and zwitterions. J Pharm Sci 95:1238–1257. 16639716 10.1002/jps.20502

[B55] Rostami-Hodjegan A (2012) Physiologically based pharmacokinetics joined with in vitro-in vivo extrapolation of ADME: a marriage under the arch of systems pharmacology. Clin Pharmacol Ther 92:50–61. 22644330 10.1038/clpt.2012.65

[B56] Rowland M Peck C Tucker G (2011) Physiologically-based pharmacokinetics in drug development and regulatory science. Annu Rev Pharmacol Toxicol 51:45–73. 20854171 10.1146/annurev-pharmtox-010510-100540

[B57] Rowland Yeo K Aarabi M Jamei M Rostami-Hodjegan A (2011) Modeling and predicting drug pharmacokinetics in patients with renal impairment. Expert Rev Clin Pharmacol 4:261–274. 22115405 10.1586/ecp.10.143

[B58] Sayama H Takubo H Komura H Kogayu M Iwaki M (2014) Application of a physiologically based pharmacokinetic model informed by a top-down approach for the prediction of pharmacokinetics in chronic kidney disease patients. AAPS J 16:1018–1028. 24912798 10.1208/s12248-014-9626-3 PMC4147047

[B59] Schnaper HW (2014) Remnant nephron physiology and the progression of chronic kidney disease. Pediatr Nephrol 29:193–202. 23715783 10.1007/s00467-013-2494-8 PMC3796124

[B60] Scotcher D Jones C Posada M Galetin A Rostami-Hodjegan A (2016 a) Key to opening kidney for in vitro-in vivo extrapolation entrance in health and disease: Part II: Mechanistic models and in vitro-in vivo extrapolation. AAPS J 18:1082–1094. 27506526 10.1208/s12248-016-9959-1

[B61] Scotcher D Jones C Posada M Rostami-Hodjegan A Galetin A (2016 b) Key to opening kidney for in vitro-in vivo extrapolation entrance in health and disease: Part I: In vitro systems and physiological Data. AAPS J 18:1067–1081. 27365096 10.1208/s12248-016-9942-x

[B62] Scotcher D Jones C Rostami-Hodjegan A Galetin A (2016 c) Novel minimal physiologically-based model for the prediction of passive tubular reabsorption and renal excretion clearance. Eur J Pharm Sci 94:59–71. 27033147 10.1016/j.ejps.2016.03.018 PMC5074076

[B63] Steiness E (1974) Renal tubular secretion of digoxin. Circulation 50:103–107. 4835256 10.1161/01.cir.50.1.103

[B64] Steiness E Waldorff S Hansen PB (1982) Renal digoxin clearance: dependence on plasma digoxin and diuresis. Eur J Clin Pharmacol 23:151–154. 7140804 10.1007/BF00545970

[B65] Tanigawara Y Okamura N Hirai M Yasuhara M Ueda K Kioka N Komano T Hori R (1992) Transport of digoxin by human P-glycoprotein expressed in a porcine kidney epithelial cell line (LLC-PK1). J Pharmacol Exp Ther 263:840–845. 1359120

[B66] Troutman MD Thakker DR (2003) Efflux ratio cannot assess P-glycoprotein-mediated attenuation of absorptive transport: asymmetric effect of P-glycoprotein on absorptive and secretory transport across Caco-2 cell monolayers. Pharm Res 20:1200–1209. 12948018 10.1023/a:1025049014674

[B67] US Food Drug Admin (2010) Guidance for Industry:Pharmacokinetics in Patients with Impaired Renal Function—Study Design, Data Analysis, and Impact on Dosing and Labeling (Revision 1), US Food and Drug Administration, Silver Spring, MD.

[B68] Varma MV Lin J Bi YA Kimoto E Rodrigues AD (2015) Quantitative rationalization of gemfibrozil drug interactions: consideration of transporters-enzyme interplay and the role of circulating metabolite gemfibrozil 1-O-β-glucuronide. Drug Metab Dispos 43:1108–1118. 25941268 10.1124/dmd.115.064303

[B69] Wagner C Zhao P Pan Y Hsu V Grillo J Huang SM Sinha V (2015) Application of physiologically based pharmacokinetic (PBPK) modeling to support dose selection: Report of an FDA public workshop on PBPK. CPT Pharmacometrics Syst Pharmacol 4:226–230. 26225246 10.1002/psp4.33 PMC4429576

[B70] Wang L Sweet DH (2013) Renal organic anion transporters (SLC22 family): expression, regulation, roles in toxicity, and impact on injury and disease. AAPS J 15:53–69. 23054972 10.1208/s12248-012-9413-y PMC3535093

[B71] Wang X Bonventre JV Parrish AR (2014) The aging kidney: increased susceptibility to nephrotoxicity. Int J Mol Sci 15:15358–15376. 25257519 10.3390/ijms150915358 PMC4200815

[B72] Zamek-Gliszczynski MJ Lee CA Poirier A Bentz J Chu X Ellens H Ishikawa T Jamei M Kalvass JC Nagar S International Transporter Consortium (2013) ITC recommendations for transporter kinetic parameter estimation and translational modeling of transport-mediated PK and DDIs in humans. Clin Pharmacol Ther 94:64–79. 23588311 10.1038/clpt.2013.45 PMC3898877

[B73] Zhang S Morris ME (2003) Effect of the flavonoids biochanin A and silymarin on the P-glycoprotein-mediated transport of digoxin and vinblastine in human intestinal Caco-2 cells. Pharm Res 20:1184–1191. 12948016 10.1023/a:1025044913766

